# Screening of Different *Physalis* Genotypes as Potential Rootstocks or Parents Against Vascular Wilt Using Physiological Markers

**DOI:** 10.3389/fpls.2020.00806

**Published:** 2020-06-12

**Authors:** Jose Luis Cháves-Gómez, Laura Marcela Becerra-Mutis, Cristhian Camilo Chávez-Arias, Hermann Restrepo-Díaz, Sandra Gómez-Caro

**Affiliations:** Departamento de Agronomía, Facultad de Ciencias Agrarias, Universidad Nacional de Colombia, Bogota, Colombia

**Keywords:** biotic stress, *Fusarium*, grafting, phenotyping, plant disease, physiological breeding

## Abstract

Cape gooseberry (*Physalis peruviana* L.) is one of the most exported Andean fruits in Colombia. Vascular wilt caused by *Fusarium oxysporum* f. sp. *physali* (FOph) has led to a reduction in crop areas in recent years. Therefore, the aim of this study was to select genotypes with resistance to vascular wilt that can be useful as rootstocks from a group of six *Physalis* genotypes (*Physalis ixocarpa*, *Physalis floridana*, and *Physalis peruviana* genotypes Colombia, Sudafrica, Peru, and Accession 62) using physiological variables such as maximum quantum efficiency of Photosystem II (Fv/Fm), leaf gas exchange properties [net photosynthesis rate (Pn) and stomatal conductance (g_*s*_)], and leaf water potential. An experiment was carried out under greenhouse conditions in which plants of the different *Physalis* materials were inoculated with the *F. oxysporum* f. sp. *physali* strain Map5 at a concentration of 1 × 10^6^ conidia mL^–1^. Physiological and disease development variables were measured at 15, 23, and 31 days after inoculation (DAI). The results obtained showed that *P. peruviana* genotypes Colombia and Sudafrica showed greater susceptibility to the disease (disease severity index 3.8 and 3.6, respectively). Net photosynthesis rate (Pn), stomatal conductance (*g*_*s*_), water potential (Ψ*_*fw*_*), and Fv/Fm ratio were lower compared to non-inoculated plants. *P. floridana* and *P. ixocarpa* plants inoculated with *F. oxysporum* showed similar behavior to non-inoculated plants for the evaluated variables. In conclusion, the results obtained suggest that these two genotypes can be considered in breeding programs or as rootstock for the establishment of cape gooseberry crops in soils with the presence of the pathogen.

## Introduction

Cape gooseberry (*Physalis peruviana* L.) is a fruit bush species from the Andean region of South America. It is of economic importance for Colombia due to the interest that international markets, mainly European countries, have shown in its fruits (Álvarez-Flórez et al., 2017; Cabrera et al., 2017). This fruit stands out for its flavor, color, and shape, as well as for its nutritional value (Valdenegro et al., 2012; Enciso-Rodríguez et al., 2013; Etzbach et al., 2018). Colombia is one of the main global producers of cape gooseberry; nevertheless, vascular wilt caused by *Fusarium oxysporum* (FO) is a disease of great economic importance for this crop since FO has caused significant yield drops (Fischer and Miranda, 2012; Osorio-Guarín et al., 2016; Simbaqueba et al., 2018). Therefore, cape gooseberry yield has decreased from 18 t. ha^–1^ in 2009 to 12.3 t. ha^–1^ in 2018 due to vascular wilt in Colombia (Agronet, 2020).

*Fusarium oxysporum* is characterized by the production of three types of propagules: macroconidia, microconidia, and chlamydospores (Okungbowa and Shittu, 2012). Plant infection and dissemination in the environment is mainly carried out by microconidia. Chlamydospores are resistance structures that allow the pathogen to remain in the soil for long periods (Zvirin et al., 2010; Zhang et al., 2015; Osorio-Guarín et al., 2016; Gordon, 2017). Besides, FO can infect plants at any development stage and its characteristic symptomatology begins with root rot, marginal and total chlorosis of mature leaves, defoliation and finally plant death (Enciso-Rodríguez et al., 2013; Joshi, 2018).

*Fusarium oxysporum* plugs vascular bundles which affects water uptake, nutrient transport, and photoassimilate distribution (Pantelides et al., 2013). On the other hand, vascular wilt causes negative physiological effects in plants such as low leaf gas exchange properties (lower photosynthetic rates and stomatal conductance) decreasing plant biomass (Nogués et al., 2002; Dong et al., 2012; Wang et al., 2015). The pathogen also causes degradation of chlorophyll pigments, reduction of chlorophyll fluorescence parameters and oxidative stress in infected plants (Morkunas and Bednarski, 2008; Dong et al., 2012; Wang et al., 2015; Chávez-Arias et al., 2019). Additionally, a low leaf water potential has been observed in infected plants, conditioning plant water status (Wang et al., 2015; Sun et al., 2017).

Disease management has been difficult due to factors such as the presence of chlamydospores in the soil, pathogen resistance to commercial fungicides and heterogeneous responses to different control strategies in cape gooseberry production areas in Colombia (Alabouvette et al., 2009; Osorio-Guarín et al., 2016). Among the most promising alternatives for disease management in the country are the use of biological control, plant breeding and grafting (rootstock selection; Simbaqueba et al., 2018). The understanding of the genetic diversity of the species and the pathosystem allow the identification of genes and traits that could contribute to plant breeding (Maryani et al., 2019). Studies on genetic diversity in the genus *Physalis* have also become important since they can provide genomic and geographic information that would facilitate breeding programs for the improvement of disease-resistant materials (Herrera et al., 2012; Chacón et al., 2016).

Physiological breeding helps to maintain or improve crop yield through the integration of morphological, agronomic and molecular traits to evaluate promising genetic resources in pre-breeding strategies (Reynolds and Trethowan, 2007; Reynolds and Langridge, 2016). The knowledge of physiological responses is a primary step to understand genetic tolerance mechanisms to stress conditions (Bhanu et al., 2016). Physiological traits such as leaf gas exchange, plant growth, leaf anatomy, leaf temperature, water relations, and chlorophyll *a* parameters have been used for plant phenotyping to abiotic stresses in different crops (Hernandez-Santana et al., 2019). Additionally, different physiological traits have been used for phenotyping of plants under abiotic and biotic stress conditions (Pandey et al., 2017). Finally, to use physiological traits for genotype selection it is necessary to consider the specific stress condition and genetic resources (Fiorani and Schurr, 2013).

Commonly used strategies for vascular wilt management have had a limited positive effect and resistant cultivars are scarce for most of the host crops the pathogen attacks (Jabnoun-Khiareddine et al., 2019). For these reasons, grafting could provide resistance to soil-borne pathogens such as fungi, bacteria, and nematodes in many crops (King et al., 2008). Among other diseases, vascular wilts caused by fungi like *Fusarium* and *Verticillium* have been the main objective of grafting since using resistant rootstocks allows planting susceptible cultivars in infested soils (King et al., 2008).

Although the mechanisms that involve grafting in plant vascular disease control are not well understood, the avoidance of the pathogen due to rootstock resistance has been reported (King et al., 2008). The increased vigor from the rootstock that allows the scion to develop even under the presence of the pathogen (Lee, 1994; Cohen et al., 2000) or the physical restriction of pathogen spread from the soil to the scion as reported by Grimault et al. (1994) could explain the lower disease levels observed when grafting is implemented. Positive results using resistant rootstocks have been reported in *Capsicum* and *Passifloraceae* species (Grech and Rijkenberg, 1991; Attia et al., 2003). Also, King et al. (2008) have studied the potential use of grafting against FO in cucumber, melon, watermelon, and tomato.

As mentioned above, *F. oxysporum* f. sp. *physali* (FOph) has infested soils, limiting crop growth, and yield in cape gooseberry (Fischer et al., 2014; Simbaqueba et al., 2018). Moreover, *P. peruviana* genotype Colombia is the most planted material in the country because of its high productivity; however, it is very susceptible to vascular wilt. Due to the low efficacy of strategies used for vascular wilt management in *P. peruviana L.* (Urrea et al., 2011), the use of rootstocks resistant to the pathogen becomes a promising alternative for this crop. Advances in cape gooseberry breeding to obtain resistance to FO are few and studies have been mainly focused on the search for genes for resistance to FOph (Enciso-Rodríguez et al., 2013; Simbaqueba et al., 2018). To evaluate different *Physalis* and other Solanaceae species as a source of resistance is of current interest in plant breeding programs. In grafting processes finding rootstocks with morphological and physiological affinity to the scions of interest becomes essential to evaluate their potential as a promising alternative to alleviate stress conditions (Zeist et al., 2017).

Grafting is one of the most extensively used techniques in the cultivation of horticultural and agronomic crops (Corso and Bonghi, 2014). The success of this technique may be influenced by several factors, such as physiological compatibility of bionts, polarity, climate or crop period, and genetic affinity between scion and rootstock combinations (Fregoni, 2005; Gregory et al., 2013). Additionally, rootstocks can be selected based on their tolerance to abiotic and biotic stress and their ability to beneficially alter scion phenotypes (Warschefsky et al., 2016). In this sense, studies on tolerance to biotic stress in species of the genus *physalis* are still limited. The available literature on these species shows that *P. peruviana* has been characterized as a possible rootstock for resistance to nematode attack (Dhivya et al., 2016).

An alternative for vascular wilt in cape gooseberry crops is to characterize *Physalis* species to select the promising ones to be used as rootstocks in pathogen-infected commercial soils. Physiological traits are an important tool that increases efficiency in breeding programs and assists in genotype selection (Pieruschka and Poorter, 2012; Reynolds and Langridge, 2016). The use of physiological markers in the *P. peruviana – F. oxysporum* f. sp. *physali* pathosystem can contribute to the selection of genotypes or rootstocks with the potential to reduce the impact of vascular wilt on cape gooseberry crops. Therefore, the aim of this study was the use of physiological variables, such as maximum quantum efficiency of Photosystem II (Fv/Fm), leaf gas exchange properties [net photosynthesis rate (Pn) and stomatal conductance (g_*s*_)], and leaf water potential, to characterize six *Physalis* genotypes (*Physalis ixocarpa*, *Physalis floridana*, and *P. peruviana* genotypes Colombia, Sudafrica, Peru, and Accession 62) as potential rootstocks for plant resistance to vascular wilt caused by FOph.

## Materials and Methods

### General Growth Conditions and Plant Material

The experiment was performed between March and April 2015 in the greenhouses of the Faculty of Agricultural Sciences at Universidad Nacional de Colombia in Bogotá (4°35′56" N, 74°04′51" W, and altitude 2557 m.a.s.l.) with a natural photoperiod of 12 h (mean photosynthetically active radiation (PAR) between 900 and 1500 μmol m^–2^ s^–1^ at noon), a 40–70% relative humidity and an average temperature of 24°C.

*Physalis peruviana* L. genotypes Colombia, Sudafrica, and Peru were selected because of their importance as the most commercialized plant material by local nurseries due to the quality of their fruit and agronomic characteristics (Herrera et al., 2012). Also, *P. peruviana* genotype Accession 62, which belongs to the germplasm collection of the central and northeastern Andean regions of Colombia, was selected for its potential resistance to FO (Osorio, 2014). On the other hand, the species *P. ixocarpa* and *Physalis floridana* were also selected because they have shown resistance to FO (Soto-Zarazúa et al., 1998; Enciso-Rodríguez et al., 2013). Finally, Table 1 summarizes the general aspects of the six evaluated *Physalis* genotypes.

**TABLE 1:** General aspects of six *Physalis* genotypes evaluated for their physiological response profile to *Fusarium oxysporum* f. sp. *physali* (FOph) as potential rootstocks or parents.

Genotypes	Origin	Common names	Response to Foph^a^	Number of chromosomes
*Physalis peruviana* Colombia Sudafrica Accession 62 Peru	South American Andes, mainly of Colombia, Peru, and Ecuador Medina, 1991	Goldenberry, aguaymanto, groselha do Peru, Kapstachelbeere, tomate silvestre, Lampion and cape gooseberry	1.33 (R) to 6.91 (S)	24, 32 and 48 with variation among genotypes Enciso-Rodríguez et al., 2013; Fischer et al., 2014
*Physalis floridana*	Southeastern United States, North America	Husk tomato	0.64 (R)	24 and 48 Liberato et al., 2014
*Physalis ixocarpa* (Syn *Physalis philadelphica)*	Central America Ramírez-Godina et al., 2013	Tomato, Mexican husk tomato, Mexican green tomato, Miltomate, tomatillo	ND	24 Ramírez-Godina et al., 2013

*^*a*^Scale of reaction to *F. oxysporum*: average scale per Accession expressed in% of damage compared to the isolate Map5 under greenhouse conditions where a value of 0–1 = % of damage 0– ≤ 5 (R = Resistant), 2–3 = ≥ 6 – ≤ 20 (PS = Slightly Susceptible), 4–5 = ≥ 21 – ≤ 60 (MS = Moderately Susceptible), 6–7 = ≥ 61 – ≤ 80 (S = Susceptible), and 8–9 = ≥ 81 – ≤ 100 (AS = Highly Susceptible); information presented for *Physalis peruviana* related to reports in Colombia (Pulido et al., 2011). ND = Not determined.*

Seeds of each plant material used were germinated in germination trays filled with peat without any nutrients (Klasmann^®^, Klasmann-Deilmann GmbH Germany) as a substrate for 51 days. When plants had two true leaves, they were fertilized every 3 days with 50 mL of a compound liquid fertilizer (Nutriponic ^®^, Walco S.A., Colombia) at a concentration of 5 mL per liter of water until the transplant date (plants with four true leaves).

### Treatment Setup

Plants from each of the selected materials were separated into two groups to establish the inoculation condition (with and without pathogen). The first group of plants was inoculated with FOph at the time of transplanting, while the remaining group was not subjected to any type of inoculation. FOph strain Map5 (highly virulent strain; Osorio-Guarín et al., 2016) was supplied by the Agricultural Microbiology Laboratory of Agrosavia (Colombia). For pathogen inoculation, conidia multiplication was performed by adding disks of Potato Dextrose Agar (PDA) culture medium with fungal mycelium to malt extract liquid medium under constant agitation (125 rpm) in a shaker (Innova 2000, New Brunswick, NJ, United States) and in a dark room at 25°C for 7 days. Then, a suspension of 1 × 10^6^ conidia mL^–1^ in sterile distilled water was adjusted (Villarreal-Navarrete et al., 2017; Chávez-Arias et al., 2019). The inoculation process was carried out 51 days after sowing (DAS) following the technique described by Lopez-Benitez et al. (2018) with modifications by dipping the roots of each of the previously washed plants in 300 ml of the conidial suspension for 3 min. Non-inoculated plants were subjected to a similar process by replacing the conidial suspension with sterile distilled water. Before inoculation, FOph absence in the plant material was confirmed by the indexation of plants according to the technique described by Leslie and Summerell (2006). Plants were then transplanted to 2 L capacity plastic pots containing a mix of soil, peat without nutrients and rice husk (3:1:1 v/v). The substrate was sterilized twice in an autoclave at 121°C and 0.103 Mpa for 25 min before transplanting.

In total, 12 treatments (six cape gooseberry genotypes vs. two inoculation conditions) were established using a completely randomized design with five repetitions per treatment. Each treatment group started with a population of 15 plants, using five individuals for each destructive evaluation of plant physiology and growth parameters at 15, 21, and 31 days after inoculation (DAI). Plants were randomly arranged in the greenhouse. Finally, the experiment lasted 81 days.

### Disease Severity Analysis and Evaluation of FOph Presence

Vascular wilt severity was calculated in each treatment every 3 days from inoculation until the end of the experiment (31 DAI). Visual evaluations of the characteristic symptoms of the disease (epinasty, chlorosis, turgor loss in leaves, and defoliation until total plant wilting) were performed using the scale proposed by Barbosa (2013). Subsequently, the disease severity index was calculated using Eq. (1) described by Chiang et al. (2017).

(1) D⁢i⁢s⁢e⁢a⁢s⁢e⁢s⁢e⁢v⁢e⁢r⁢i⁢t⁢y⁢I⁢n⁢d⁢e⁢x=(∑(n⁢v)/V)

where *n* is the infection level according to the scale, *v* is the number of plants in each level and *V* is the total number of evaluated plants.

Finally, the disease intensity through the area under the disease progress curve (AUDPC) was estimated in each treatment following the trapezoidal integration method (Campbell and Madden, 1990) as presented in Eq. (2):

(2) $$A\,U\,D\,P\,C=\left\{\sum_{\text{i}=1}^{n-1}\left[\left(y_{i}+y_{i+1}\right)/2\right]*\left(t_{i+1}-t_{i}\right)\right\}$$

where *n* is the number of evaluations, *y*_*i*_ and *y*_*i+1*_ are the values of the severity scale on each evaluation date and (*t*_*i* + 1_−*t*_*i*_) is the time interval between evaluations.

On the other hand, FOph presence in the different genotypes was determined by obtaining typical colonies of the pathogen from explants taken from the base of the stem of inoculated and non-inoculated plants in PDA medium at 25°C (Leslie and Summerell, 2006) at 15, 23, and 31 DAI. Likewise, the isolation frequencies were calculated as shown in Eq. (3) to determine the infection and establishment of the pathogen in each of the genotypes (Šišić et al., 2018):

(3) $$F\,r\,e\,q\,u\,e\,n\,c\,y\,o\,f\,i\,s\,o\,l\,a\,t\,i\,o\,n=\frac{n}{N}$$

where *n* is the number of stem explants infected with the pathogen and *N* is the total number of stem explants analyzed in each genotype.

### Leaf Gas Exchange Properties and Maximum Quantum Efficiency of PSII (Fv/Fm)

Fully expanded leaves from the middle third of the plant were selected and dark-adapted for 15 min. Then, they received an actinic light pulse of up to 2.600 μmol m^–2^ s^–1^ on the surface to obtain the Fv/Fm ratio. The net photosynthesis (Pn) and leaf stomatal conductance (g_*s*_) were also estimated on the same leaves using a portable photosynthesis meter (Li-Cor 6200, Li-Cor Inc., Lincoln, NE, United States). The conditions of the chamber during leaf gas exchange measurements were: PAR 500–1200 μmol m^–2^ s^–1^ at noon; leaf temperature 27°C ± 2 and a CO_2_ concentration of 400 ± 15 μmol mol^–1^. Finally, intrinsic water use efficiency (WUE*_*i*_*) was also calculated as the Pn/g_*s*_ ratio. The measurements were performed between 10:00 and 14:00 on completely sunny days.

### Screening for FOph Tolerance in Terms of the Fv/Fm Ratio

The Fv/Fm readings recorded at the second sampling point (23 DAI) were used to calculate the decrease in the maximum quantum efficiency of PSII (DQE), since plants of some genotypes died due to FOph inoculation at 31 DAI. DQE was calculated by the (Eq. 4):

(4) DQE=[(Fv/FmCP-(Fv/FmIP)/(Fv/FmCP)]×100

where CP and IP represent control and inoculated plants, respectively.

Then, the cape gooseberry genotypes were ranked into four categories, explained as follows: DQE ≤ 25, high tolerance; between 26 and 41, moderate tolerance; between 42 and 55, low tolerance; and ≥56, susceptible.

### Leaf Water Potential

The leaves used for Fv/Fm, Pn, and g_*s*_ readings were cut to record the leaf water potential (Ψ*_*wf*_*) using a Schollander pressure chamber (PMS, Model 615, OR, United States) at midday.

### Growth Parameters and Relative Chlorophyll Concentration

Leaves, stems, and roots from each of the plants of the different genotypes were collected, weighed, and dried separately at 80°C. Additionally, the relative chlorophyll content was estimated in SPAD units using a chlorophyll meter (SPAD 502-Konica, Minolta Sensing Inc., Osaka, Japan) on the same leaves used for Fv/Fm, Pn, and g_*s*_ readings. Finally, all physiological variables previously described were recorded at 15, 23, and 31 DAI.

### Relative Tolerance Index (RTI)

The relative tolerance index was calculated indirectly to determine the tolerance of *Physalis* genotypes to FOph inoculation, using the net photosynthesis (Pn) of the inoculated genotypes in relation to the control genotypes (without inoculation). The RTI was obtained at 23 DAI, using (Eq. 5) adapted from Chávez-Arias et al. (2020):

(5) R⁢T⁢I=(P⁢n⁢i⁢n⁢o⁢c⁢u⁢l⁢a⁢t⁢e⁢d⁢g⁢e⁢n⁢o⁢t⁢y⁢p⁢e/P⁢n⁢n⁢o⁢n-i⁢n⁢o⁢c⁢u⁢l⁢a⁢t⁢e⁢d⁢g⁢e⁢n⁢o⁢t⁢y⁢p⁢e)×100

### Experimental Design and Data Analysis

Data were analyzed using a factorial arrangement in which the main factor was the inoculation condition (with or without FOph) and the second one was the genotypes. Each treatment consisted of 5 plants per repetition. An analysis of variance (ANOVA) was also performed and when significant differences (*P* ≤ 0.05) were found, a Tukey *post hoc* test was used for mean comparison. Likewise, a correlation analysis between RTI and AUDPC, or Fv/Fm was carried out to determine the best genotype under inoculation conditions. The percentage values were transformed using the arcsine function. Data were analyzed using the software Statistix v 9.0 (Analytical Software, Tallahassee, FL, United States). SigmaPlot (version 10.0; Systat Software, San Jose, CA, United States) was used to draw figures, the three-dimensional plot, and to perform the correlation analysis.

## Results

### Disease Severity

**FIGURE 1:**
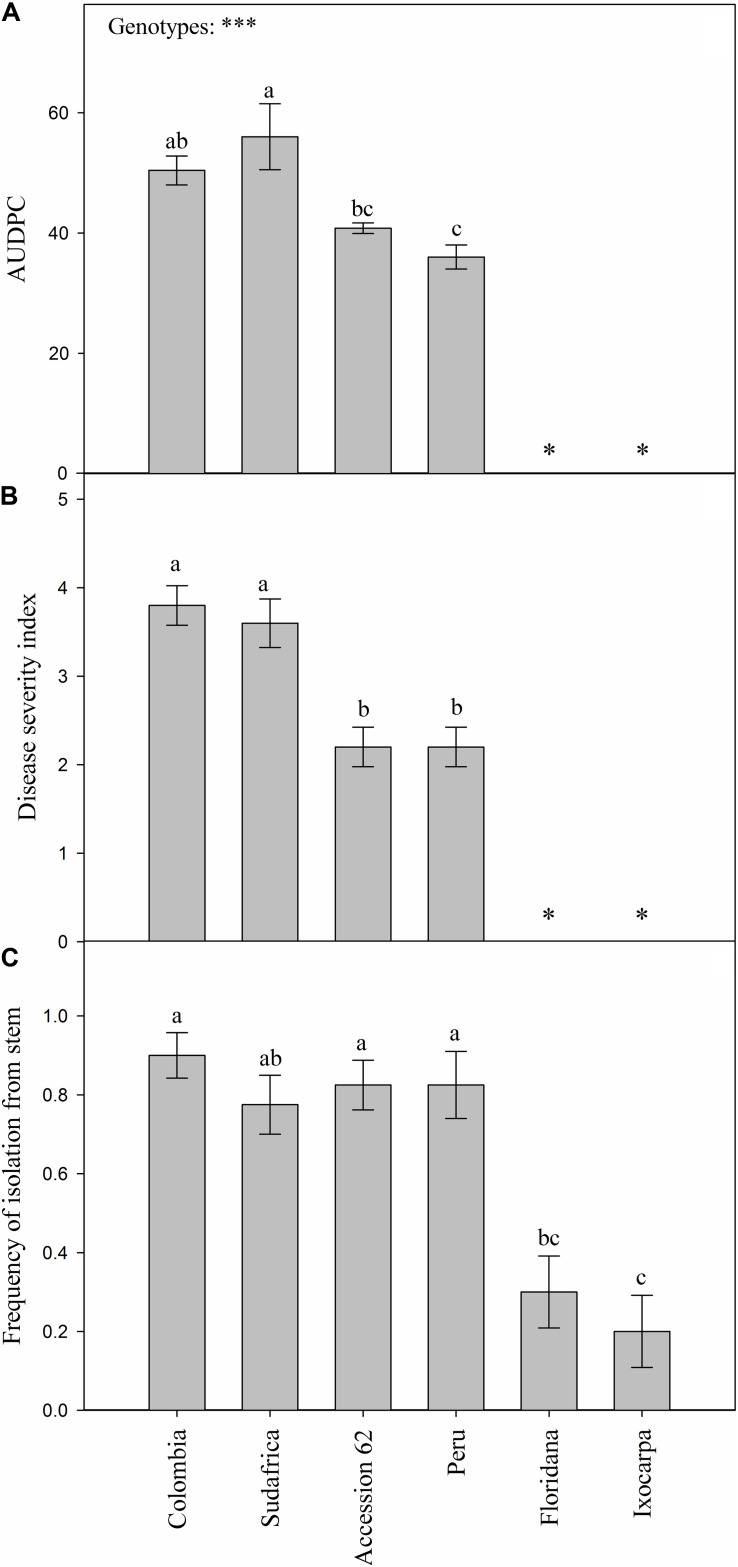
Area under the disease progress curve (AUDPC; **A)** and disease severity index **(B)** of vascular wilt caused by *Fusarium oxysporum* f. sp. *physali* (FOph) and frequency of pathogen isolation from plant stems **(C)** of six *Physalis* genotypes [Colombia, Sudafrica, Accession 62, Peru, *Physalis floridana* (Floridana), and *Physalis ixocarpa* (Ixocarpa)] at 31 days after inoculation (DAI). Each column represents the mean of five data ± standard error (*n* = 5). Bars followed by different letters indicate statistically significant differences according to the Tukey test (*P* ≤ 0.05). ^∗^Means that plants of genotypes Ixocarpa and Floridana are materials that did not show any visual symptoms of the disease. *p*-Values of the ANOVA of genotypes are indicated as ^∗∗∗^*p* < 0.001.

**FIGURE 2:**
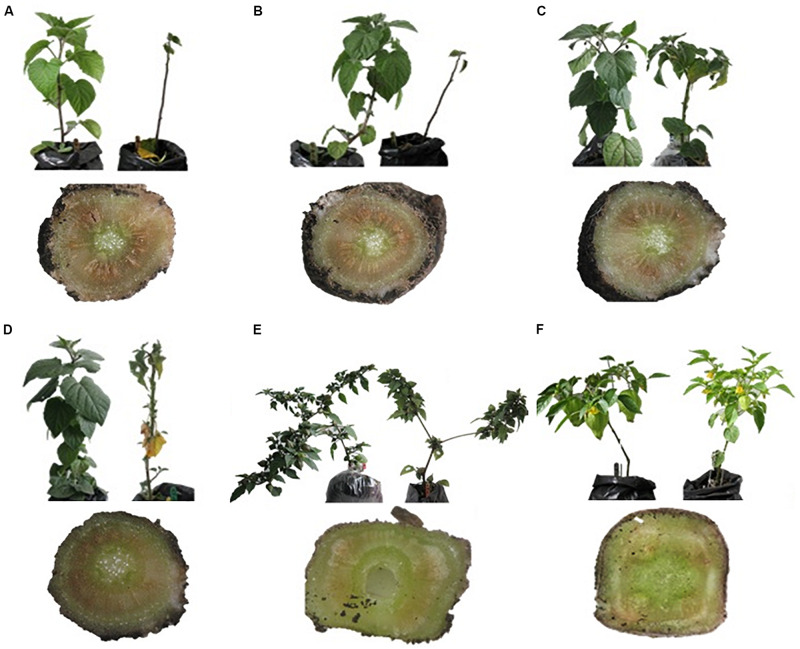
Leaf wilting and vascular browning of six *Physalis* genotypes with and without *Fusarium oxysporum* f. sp. *physali* (FOph). **(A)** Colombia, **(B)** Sudafrica, **(C)** Accession 62, **(D)** Peru, **(E)** *Physalis floridana*, and **(F)** *Physalis ixocarpa* at 23 days after inoculation (DAI). **(A–C)** Materials of interest for commercial production; **(D–F)** genotypes with rootstock potential. For each genotype, control (left) and FOph-inoculated (right) plants are shown in the upper part of the image; lower part of the image: cross-section of plants inoculated with the pathogen showing the intensity of the damage at the vascular level.

FOph-inoculated plants of all the different evaluated genotypes showed typical disease symptoms. Differences (*P* ≤ 0.001) were observed on the AUDPC and disease severity index in the different genotypes at 31 DAI. AUDPC showed the highest values in the plants of genotypes Sudafrica (56.0) and Colombia (50.4; Figure 1A). Intermediate values were recorded in “Accession 62” and “Peru” plants (40.8 and 36.0, respectively; Figure 1A), while plants of genotypes *P. ixocarpa*, and *P. floridana* were visually asymptomatic and did not present any disease value (Figure 1A). Likewise, similar trends were also obtained on the disease severity index at 31 DAI (Figure 1B). On the other hand, the frequency of pathogen isolation in PDA was lower in genotypes *P. ixocarpa* (0.20), and *P. floridana* (0.30) in contrast to all the other evaluated genotypes. In this case, “Colombia” (0.90), “Peru,” and “Accession 62” (0.83 both), and “Sudafrica” (0.78) showed higher FOph isolation values but without significant differences among them (Figure 1C). In this sense, the presence of the pathogen in inoculated plants was also confirmed by isolation in PDA from affected plant material (Figure 2). No pathogen was isolated from non-inoculated plants used as controls.

### Leaf Gas Exchange Properties (Leaf Photosynthesis and Stomatal Conductance), Fv/Fm Ratio and SPAD Readings

**FIGURE 3:**
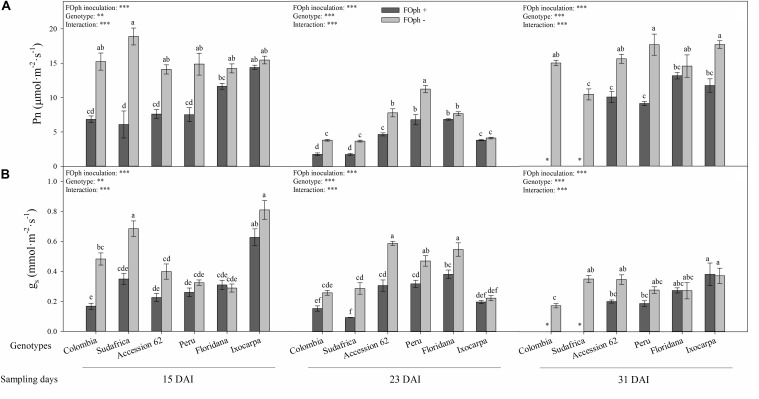
Photosynthesis (Pn; **A)** and stomatal conductance (g_*s*_; **B)** of six cape gooseberry genotypes [Colombia, Sudafrica, Accession 62, Peru, *Physalis floridana* (Floridana), and *Physalis ixocarpa* (Ixocarpa)] with (dark gray bars) or without (light gray bars) *Fusarium oxysporum* f. sp. *physali* (FOph) inoculation at three different sampling points [15, 23, and 31 days after inoculation (DAI)]. Each column represents the mean of five data ± standard error (*n* = 5). Bars followed by different letters indicate statistically significant differences according to the Tukey test (*P* ≤ 0.05). ^∗^Means that the photosynthesis and stomatal conductance were not determined due to the death of the genotype because of the pathogen. *p*-Values of the ANOVA of genotypes, FOph inoculation and their interaction are indicated as ^∗∗^*p* < 0.01, and ^∗∗∗^*p* < 0.001.

**FIGURE 4:**
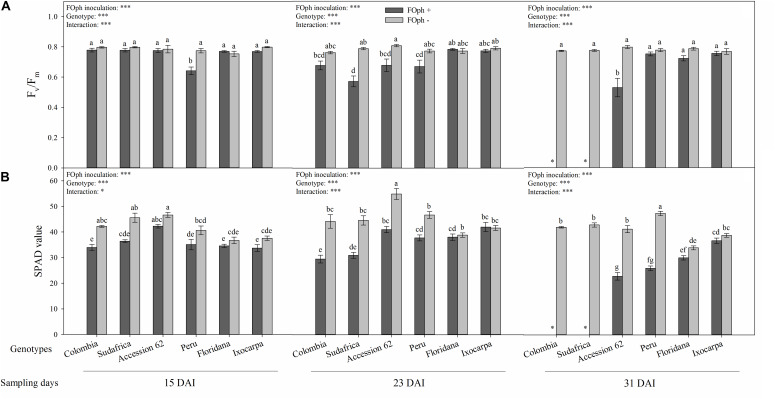
Efficiency of photosystem II (PSII; Fv/Fm ratio; **A**) and relative chlorophyll content (SPAD value; **B**) of six cape gooseberry genotypes [Colombia, Sudafrica, Accession 62, Peru, *Physalis floridana* (Floridana), and *Physalis ixocarpa* (Ixocarpa)] with (dark gray bars) or without (light gray bars) *Fusarium oxysporum* f. sp. *physali* (FOph) inoculation at three different sampling points [15, 23, and 31 days after inoculation (DAI)]. Each column represents the mean of five data ± standard error (*n* = 5). Bars followed by different letters indicate statistically significant differences according to the Tukey test (*P* ≤ 0.05). ^∗^Means that the efficiency of PSII and relative chlorophyll content were not determined due to the death of the genotype because of the pathogen. *p*-Values of the ANOVA of genotypes, FOph inoculation and their interaction are indicated as ^∗^*p* < 0.05 and ^∗∗∗^*p* < 0.001.

Differences (*P* ≤ 0.01) in the interaction between genotypes and FOph inoculation on net photosynthesis (Pn), leaf stomatal conductance (*g*_*s*_), Fv/Fm ratio, and SPAD readings are shown in Figures 3, 4. At 15 DAI, inoculated genotypes started to show a reduction in Pn compared to non-inoculated plants, except for genotypes *P. ixocarpa* (14.4 mmol cm^–2^ s^–1^) and *P. floridana* (11.6 mmol cm^–2^ s^–1^), which showed similar values to non-inoculated plants (*P. ixocarpa* 15.5 mmol cm^–2^ s^–1^ and *P. floridana* 14.2 mmol cm^–2^ s^–1^). At the second sampling point (23 DAI), genotypes Colombia, Sudafrica, Accession 62 and Peru inoculated with FOph continued with a low Pn in comparison with *P. floridana* and *P. ixocarpa*, which did not show differences between inoculation conditions. At the end of the experiment (31 DAI), leaf photosynthesis could not be recorded in plants of genotypes Colombia and Sudafrica because this group of plants died due to vascular wilt. Meanwhile, FOph-inoculated plants of genotypes *P. ixocarpa*, Accession 62, and Peru showed a reduction (∼40%) in Pn. Finally, genotype *P. floridana* plants did not show any differences in Pn as a result of FOph inoculation (inoculated (13.2 mmol cm^–2^ s^–1^) vs. non-inoculated (14.6 mmol cm^–2^ s^–1^) plants; Figure 3A).

Statistical differences (*P* ≤ 0.01) were also found for g_*s*_ in the interaction among genotypes, and inoculation at each sampling point. At 15 DAI, a reduction in g_*s*_ was observed in FOph-inoculated plants of genotypes Colombia, *P. ixocarpa*, Sudafrica and Accession 62. However, inoculated *P. floridana* (0.31 mmol cm^–2^ s^–1^), *P. ixocarpa* (0.63 mmol cm^–2^ s^–1^), and “Peru” (0.26 mmol cm^–2^ s^–1^) plants did not show any differences compared to their control plants (0.28 mmol cm^–2^ s^–1^, 0.81 cm^–2^ s^–1^, and 0.32 mmol cm^–2^ s^–1^, respectively). AT 23 DAI, gs continued to show similar behavior to the one registered in the previous sampling point. At 31 DAI (end of the experiment), genotypes Accession 62 and Peru showed lower g_*s*_ values in plants affected by vascular wilt; however, FOph inoculation did not cause any change on gs in genotypes *P. floridana* and *P. ixocarpa* (Figure 3B).

The Fv/Fm ratio showed similar results for the diseased and healthy plants of genotypes Colombia, *P. ixocarpa*, Sudafrica, Accession 62, and *P. floridana* at 15 DAI. However, plants with vascular wilt of genotype Peru showed a decrease (∼23%) compared to their control. At the second sampling point (23 DAI), the effect of FOph inoculation on the Fv/Fm ratio began to be observed, with genotypes Colombia (0.68), Sudafrica (0.57), Peru (0.67), and Accession 62 (0.68) showing a slight decrease in this variable compared to their control plants (cape gooseberry plants without FOph; ∼0.76); however, genotypes *P. ixocarpa*, and *P. floridana* did not show differences on Fv/Fm ratio between inoculation conditions. At 31 DAI, these values continued to be similar in genotypes *P. ixocarpa* and *P. floridana* under both inoculation conditions. Nevertheless, “Peru” plants with FOph showed an increase in the Fv/Fm with values similar to those registered in the plants of their respective control compared to the previous sampling point. Finally, “Accession 62” plants with vascular wilt showed a reduction (∼33%) of these values compared to plants without the pathogen (Figure 4A).

The relative chlorophyll content (SPAD units) registered a reduction in plants with vascular wilt in all genotypes at 15 DAI. Then, this trend was kept at 23 DAI. At the last sampling point (31 DAI), “Accession 62” (22.8), and “Peru” (25.9) plants with FOph inoculation continued to show a reduction in the relative chlorophyll content in SPAD units compared to their control plants (41.1 and 47.2, respectively). Finally, plants with and without FOph from *P. ixocarpa* and *P. floridana* did not show any changes in the SPAD readings (Figure 4B).

### Biomass Yield

**FIGURE 5:**
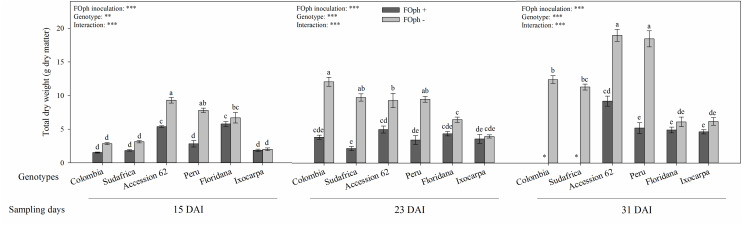
Total dry weight of six cape gooseberry genotypes [Colombia, Sudafrica, Accession 62, Peru, *Physalis floridana* (Floridana), and *Physalis ixocarpa* (Ixocarpa)] with (dark gray bars) or without (light gray bars) *Fusarium oxysporum* f. sp. *physali* (FOph) inoculation at three different sampling points [15, 23, and 31 days after inoculation (DAI)]. Each column represents the mean of five data ± standard error (*n* = 5). Bars followed by different letters indicate statistically significant differences according to the Tukey test (*P* ≤ 0.05). ^∗^Means that the total biomass was not determined due to the death of the genotype because of the pathogen. *p*-Values of the ANOVA of genotypes, FOph inoculation and their interaction are indicated as ^∗∗∗^*p* < 0.001.

Total dry weight (TDW) was also influenced by the interaction between genotypes and the inoculation condition at the different sampling points (*P* ≤ 0.001; Figure 5). The TDW registered a decrease in plants with vascular wilt in genotypes Colombia (1.62 *g* dry matter), Sudafrica (1.84 *g* dry matter), Accession 62 (5.39 *g* dry matter), and Peru (2.82 g dry matter) compared to their respective controls (2.84 *g* dry matter, 3.14 *g* dry matter, 9.31 *g* dry matter, and 7.81 *g* dry matter, respectively). On the other hand, inoculated and non-inoculated plants of genotypes *P. ixocarpa* and *P. floridana* showed similar values in their TDW at 15 DAI. At 23 DAI, a drop in biomass accumulation was mainly observed in genotypes Colombia, Sudafrica, and Peru. At 31 DAI, TDW continued to be lower in “Accession 62” and “Peru” plants (∼51% and ∼71%, respectively) compared to their controls. Additionally, a decrease in TDW was observed in diseased plants compared to the healthy ones in genotypes *P. floridana and P. ixocarpa* (∼25%).

### Leaf Water Potential

**FIGURE 6:**
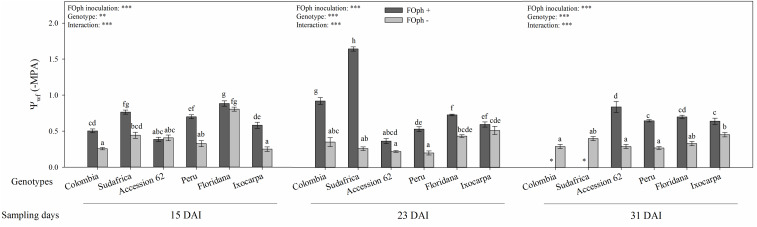
Leaf water potential (Ψ*_*wf*_*) of six cape gooseberry [Colombia, Sudafrica, Accession 62, Peru, *Physalis floridana* (Floridana), and *Physalis ixocarpa* (Ixocarpa)] with (dark gray bars) or without (light gray bars) *Fusarium oxysporum* f. sp. *physali* (FOph) inoculation at three different sampling points [15, 23, and 31 days after inoculation (DAI)]. Each column represents the mean of five data ± standard error (*n* = 5). Bars followed by different letters indicate statistically significant differences according to the Tukey test (*P* ≤ 0.05). ^∗^Means that the leaf water potential was not determined due to the death of the genotype because of the pathogen. *p*-Values of the ANOVA of genotypes, FOph inoculation and their interaction are indicated as ^∗∗∗^*p* < 0.001.

Leaf water potential (Ψ*_*wf*_*) was also influenced by the inoculation factor in all the evaluated genotypes (*P* ≤ 0.001; Figure 6). At 15 DAI, all genotypes without FOph inoculation showed, in general, a better Ψ*_*wf*_* than FOph-plants. At 23 DAI, it could be observed that FOph negatively affected Ψ*_*wf*_* in genotypes Colombia (−0,9 MPa) and Sudafrica (−1.6 MPa) compared to Accession 62 (−0.35 MPa), Peru (−0.55 MPa), *P. ixocarpa* (−0.7 Mpa), and *P. floridana* (−0.72 MPa; Figure 6). At 31 DAI, the plants of genotypes Colombia and Sudafrica could not be evaluated because they also died of vascular wilt at 27 DAI. On the other hand, a decrease in Ψ*_*wf*_* was observed in the remaining genotypes [*P. ixocarpa* (−0.60 Mpa), Accession 62 (−0.79 Mpa), *P. floridana* (−0.68 Mpa), and Peru (−0.59 Mpa)] due to FOph inoculation compared to their control plants [*P. ixocarpa* (−0.60 Mpa), “Accession 62” (−0.79 Mpa), *P. floridana* (−0.68 Mpa), and “Peru” (−0.59 Mpa).

### Screening for FOph Tolerant Genotypes

**FIGURE 7:**
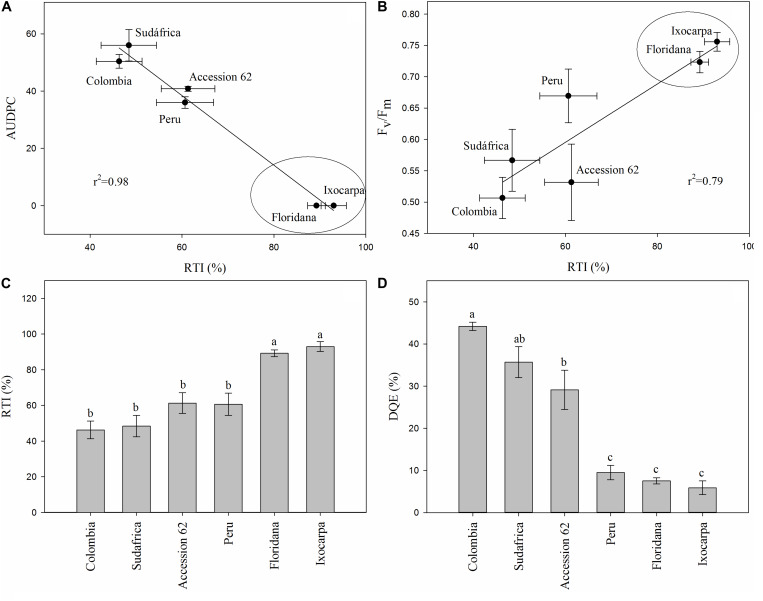
Correlation between the area under the disease progress curve (AUDPC; **A**) or the Fv/Fm ratio **(B)** and the relative tolerance index (RTI; **C**) and the decrease in the maximum efficiency of PSII (DQE; **D**) in six cape gooseberry genotypes [(Colombia, Sudafrica, Accession 62, Peru, *Physalis floridana* (Floridana) and *Physalis ixocarpa* (Ixocarpa)] inoculated with *Fusarium oxysporum* f. sp. *physali* (FOph) at 23 days after inoculation (DAI). Data represents the mean of five data ± standard error (*n* = 5). Bars followed by different letters indicate statistically significant differences according to the Tukey test (*P* ≤ 0.05).

**FIGURE 8:**
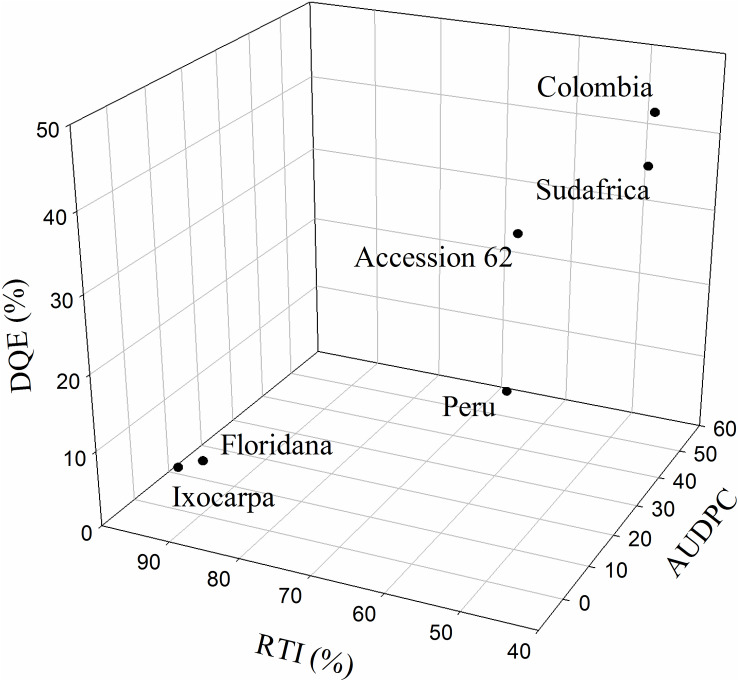
Three-dimensional plot [area under the disease progress curve (AUDPC), decrease in the maximum efficiency of PSII (DQE), and relative tolerance index (RTI)] for six cape gooseberry genotypes [Colombia, Sudafrica, Accession 62, Peru, *Physalis floridana* (Floridana), and *Physalis ixocarpa* (Ixocarpa)] inoculated with *Fusarium oxysporum* f. sp. *physali* (FOph) at 23 days after inoculation (DAI). Data represents the mean of five data ± standard error (*n* = 5).

The RTI, the correlations between RTI and AUDPC or Fm/Fv and the decrease in the maximum efficiency of PSII (DQE) were calculated only at 23 DAI to detect cape gooseberry genotypes as possible rootstock with some degree of resistance to FOph (Figure 7). The RTI and its respective correlations with the AUDPC (*r*^2^ = 0.98) and Fv/Fm ratio (*r*^2^ = 0.79) showed that genotypes *P. floridana* and *P. ixocarpa* had lower disease development and better efficiency of PSII. This was reflected in a higher RTI (90% for both genotypes) under FOph inoculation conditions (Figures 7A–C). The previous observations were also corroborated by the DQE since this index showed that FOph had less effect on the efficiency of PSII of the previously mentioned genotypes with DQE values of 25, classifying them with a good level of tolerance to the stress conditions caused by FOph (Figure 7D and Table 2). Finally, the three-dimensional plot (AUDPC, RTI, and DQE) also corroborated the conducted correlation analysis, with genotypes *P. floridana* and *P. ixocarpa* showing a better physiological behavior (less affectation on Pn and Fv/Fm ratio) under FOph infection (Figure 8).

**TABLE 2:** Classification of *Physalis* genotypes based on the decrease in the maximum quantum efficiency of photosystem II (Fv/Fm ratio; DQE) under *Fusarium oxysporum* f. sp. *physali* (FOph) at 23 days after inoculation (DAI).

DQE (%)^z^	Classification	Studied genotypes
DQE ≤ 25	Good tolerance	Ixocarpa, Floridana, Peru
26 ≥ DQE ≤ 41	Moderate tolerance	Sudafrica, Accession 62
42 ≥ DQE ≤ 55	Low tolerance	Colombia
DQE ≥ 56	Sensitive	–

*^*z*^DQE – decrease in the maximum quantum efficiency of photosystem II (Fv/Fm ratio) with FOph inoculated genotypes compared non-inoculated genotypes.*

## Discussion

Rootstocks are an important component in modern fruit production because of their ability to acclimate a particular crop to diverse environmental conditions and their capability to provide traits that are absent in the scion, such as disease resistance (Gainza et al., 2015). In this sense, the knowledge of mechanisms of resistance to vascular wilt in *Physalis* species and their use as rootstocks are not well known. Therefore, the effect of FOph inoculation on different *Physalis* genotypes (*P. ixocarpa*, Sudafrica, Colombia, Accession 62, *P. floridana*, and Peru) was evaluated at the physiological and pathological levels in the present work to identify genotypes with potential as rootstocks to mitigate the negative effect of FOph in cape gooseberry crops.

The physiological response of rootstocks under stress conditions helps to identify ideal genotypes to face challenges in fruit crops (Nimbolkar et al., 2016). A limitation of leaf Pn due to partial stomatal closure has been described as an early response to FO infection (Nali et al., 2011). In this regard, the negative impact of FOph on leaf gas exchange properties (Pn and gs) was a characteristic response of plant (genotypes Colombia, *P. ixocarpa*, Sudafrica, Accession 62, and Peru) susceptibility to vascular wilt. Low Pn and *g*_*s*_ values have also been recorded in studies performed by Wang et al. (2015) and Sun et al. (2017) in cucumber plants inoculated with *F. oxysporum* f. sp. *cucumerinum*. These authors report that the infection of the pathogen inhibits the photosynthetic capacity because it induces stomatal closure and decreases the intracellular CO_2_ concentration, affecting carboxylation efficiency and Rubisco regeneration. On the other hand, diseased plants of genotype *P. floridana* did not register any reduction of Pn and *g*_*s*_ compared to its control plants. Screenings carried out by Ahmad et al. (2010) in citrus rootstocks found that C-35 Citrange (*Ruby orange* × *Poncirus trifoliate*) and Cox mandarin hybrid (*Scarlet mandarin* × *Poncirus trifoliate*) did not show any change in their leaf gas exchange properties. The little variation of Pn in genotype *P. floridana* under FOph inoculation conditions may be associated with an increased N metabolism activity. Zhou et al. (2017) concluded that higher NO_3_^–^ nutrition increased cucumber resistance to vascular wilt by suppressing FO colonization and fusaric acid production, which increases plant tolerance. The previous statement also helps to understand why the most tolerant genotypes (*P. floridana* and *P. ixocarpa*) to FOph showed less variation in their relative chlorophyll content (SPAD units).

The Fv/Fm ratio is a classic parameter that reflects the whole PSII function, and its decrease is associated with PSII damage under stress conditions (Penella et al., 2013). In the present study, the Fv/Fm values were seriously reduced by FOph inoculation in genotypes Accession 62, Peru, and Sudafrica. These results are consistent with findings by Pshibytko et al. (2006), who also reported a significant reduction in Fv/Fm levels of tomato plants due to FO (healthy plants 0.808 vs. FO-infected plants 0.719). Additionally, these authors mention that *Fusarium* causes a reduction of Fv/Fm due to an inhibition of the photosynthetic activity. Nogués et al. (2002) also stated that FO infection may cause a down-regulation of electron transport and photodamage to PSII reaction centers as a secondary effect of the depressed CO_2_ assimilation of leaves. Therefore, susceptible *Physalis* genotypes such as Accession 62, Colombia, or Peru showed lower Fv/Fm and chlorophyll content values. In contrast, *P. floridana* and *P. ixocarpa* had lower damage in the PSII centers (Figure 4A), suggesting that the Fv/Fm ratio is an easy and non-destructive method to screen photosynthesis damage under stressful conditions.

The leaf water potential is also an important indicator of plant water status (Kakani et al., 2007). In the present research, the negative effect on the plant water status generated by vascular wilt was recorded for all evaluated genotypes. Similar results were obtained in cape gooseberry plants of the genotype Colombia inoculated with FOph (healthy plants −0.19 Mpa vs. FOph-infected plants −0.52 Mpa; Chávez-Arias et al., 2019). A low Ψ*_*wf*_* is one of the main responses to the stress condition caused by FO, in which the main factors that affect the plant water status are the uncontrolled loss of water due to cell membrane damage, the production of toxins by the pathogen and vascular plugging (Nogués et al., 2002; Dong et al., 2012; Wang et al., 2015; Sun et al., 2017).

The alterations caused by vascular wilt on the leaf gas exchange properties and Ψ*_*wf*_* had a negative effect on total biomass accumulation (TDW) in most of the evaluated genotypes, except for *P. floridana* plants. Giurgiu et al. (2018) and Chávez-Arias et al. (2019) also recorded a reduction in biomass accumulation with periods of FO inoculation greater than 15 days in St. John’s wort and cape gooseberry plants, respectively, with a reduction of water and nutrient uptake and an indirect decrease of the synthesis of photosynthetic pigments (Pantelides et al., 2013).

Regarding vascular wilt development, genotypes Colombia and Sudafrica showed the highest values of disease severity index, AUDPC, and frequency of FOph isolation followed by Peru and Accession 62 plants. However, genotypes *P. ixocarpa* and *P. floridana* did not show the characteristic symptoms of the disease during the evaluated period although plants were colonized by the pathogen. As a result, FOph was isolated from inoculated plants of these two genotypes in PDA media at low frequencies (Figure 2C). The positive response observed in these two genotypes (*P. ixocarpa* and *P. floridana*) after FOph inoculation is of remarkable importance taking into account that the Map5 isolate used is highly virulent and caused plant death of the highly susceptible genotypes (Colombia and Sudafrica) in the middle of the evaluation time. This study confirms previous observations that suggested that these species showed resistance to FO, possibly due to their characteristics of genetic diversity (Apodaca-Sánchez et al., 2004; Pulido et al., 2011; Liberato et al., 2014).

Although not evaluated in this study, the results observed in less affected genotypes (low AUPC, low DEQ, or high RTI) may be related to early defense responses associated with xylem occlusion by compounds or parenchymal tissue growth that can limit pathogen growth. Plant defense mechanisms against vascular wilts may involve not only chemical defense responses that inhibit pathogen growth but also physical defense mechanisms that restrict it from further spread in the xylem vessels (Yadeta and Thomma, 2013). Occluding material in the xylem vessels of resistant cultivars or with some level of resistance has been observed in bean genotypes as a reaction against *F. oxysporum* f. sp. *phaseoli* infection (Pereira et al., 2013). Tyloses are also reported among the structural alterations in the host tissue that block the spread of vascular pathogens due to the growth of vessel-associated parenchyma cells in the xylem (Agrios, 2005; Fradin and Thomma, 2006; Yadeta and Thomma, 2013).

In summary, vascular wilt caused by FOph inhibits the photosynthesis in cape gooseberry plants and different mechanisms are involved in the suppression of the photosynthetic activity depending on the genotype and the development of the pathogen. In this regard, the Fv/Fm ratio was an indicator of the differentiation of plant response to vascular wilt between genotypes. The findings of the current study may also suggest Pn, gs, Fv/Fm ratio, and TDW to be considered as variables of interest in the phenotyping of *Physalis* genotypes for resistance to FOph. It was also confirmed that genotypes Colombia and Sudafrica are highly susceptible to FOph inoculation, while *P. ixocarpa* and *P. floridana* had a better physiological response to the pathogen infection and the subsequent development of the disease. Since FOph infests most of the soils of production areas in Colombia, grafting *P. peruviana* genotypes on resistant *Physalis* rootstocks may contribute to sow cape gooseberry crops again in those abandoned infested areas. Therefore, genotypes *P. ixocarpa* and *P. floridana* can be suggested as candidates to either enter a breeding program for vascular wilt management or to be used as rootstocks in future studies pursuing a real integrated approach for the management of this soil-borne disease.

## Data Availability Statement

The original contributions presented in the study are included in the article, further inquiries can be directed to the corresponding author.

## Author Contributions

CC-A, HR-D, and SG-C: conceptualization and Writing – review and editing. JC-G and LB-M: methodology, investigation, and writing – original draft. JC-G, LB-M, CC-A, HR-D, and SG-C: validation. JC-G, LB-M, JC-G, and LB-M: formal analysis. JC-G, LB-M, and CC-A: data curation. HR-D and SG-C: resources, supervision, project administration, and funding acquisition. All authors agreed to be accountable for the content of the work.

## Conflict of Interest

The authors declare that the research was conducted in the absence of any commercial or financial relationships that could be construed as a potential conflict of interest.
